# Dual role of neutrophils in modulating liver injury and fibrosis during development and resolution of diet-induced murine steatohepatitis

**DOI:** 10.1038/s41598-021-03679-w

**Published:** 2021-12-17

**Authors:** Andrea D. Kim, Sung Eun Kim, Aleksandra Leszczynska, Benedikt Kaufmann, Agustina Reca, Dong Joon Kim, Ariel E. Feldstein

**Affiliations:** 1 Department of Pediatrics, University of California San Diego, La Jolla, USA; 2 Department of Internal Medicine, Hallym University College of Medicine, Chuncheon, Republic of Korea; 3 Institute for Liver and Digestive Diseases, Hallym University, Chuncheon, Republic of Korea; 4 Department of Surgery, TUM School of Medicine, Klinikum rechts der Isar, Technical University of Munich, Munich, Germany

**Keywords:** Gastroenterology, Medical research

## Abstract

Inflammatory changes in the liver represent a key feature of non-alcoholic steatohepatitis (NASH), the progressive form of non-alcoholic fatty liver disease (NAFLD). Innate immune activation including hepatic neutrophilic infiltration acts as an important inflammatory trigger as well as a potential mediator of inflammation resolution. In this study, we dissected the effects of neutrophil depletion via anti-lymphocyte antigen 6 complex locus G6D (Ly6G) antibodies administration during ongoing high fat-fructose-cholesterol (FFC) diet-induced murine NASH and during inflammation resolution by switching into a low-fat control diet. During NASH progression, protective effects were shown as HSC activation, cell infiltration and activation of pro-inflammatory macrophages were ameliorated. Furthermore, these changes were contrasted with the effects observed when neutrophil depletion was performed during the resolution phase. Impaired resolving mechanisms, such as a failure to balance the pro and anti-inflammatory cytokines ratio, deficient macrophage phenotypic switch into a pro-restorative profile, and defective repair and remodeling processes were observed when neutrophils were depleted in this scenario. This study described phase-dependent contrasting roles of neutrophils as triggers and pro-resolutive mediators of liver injury and fibrosis associated with diet-induced NASH in mice. These findings have important translational implications at the time of designing NASH therapeutic strategies.

## Introduction

Non-alcoholic fatty liver disease (NAFLD) is currently the most common chronic liver disease in Western countries. It represents a wide spectrum of disease with non-alcoholic steatohepatitis (NASH) being the progressive form, eventually leading to the development of liver cirrhosis and hepatocellular carcinoma. The central histopathological features of NASH consist of hepatocellular damage, inflammation and various degrees of fibrosis^1,2^. Enhanced infiltration of neutrophils is a key component of the inflammatory changes observed in livers from humans and mice with NASH. Growing evidence supports the concept of neutrophils as important mediators of the transition from steatosis to NASH^3^ via mechanisms that include the release of granules with proteolytic activity and reactive oxygen species, engulfment of microorganisms, and formation of neutrophil traps^3,4^. While the specific mechanisms through which neutrophils engage in worsening of liver inflammation have been thoroughly studied, their contribution to abnormal wound healing responses and development of liver fibrosis remain only partially understood. Additionally, neutrophils have been shown to be highly sophisticated immune cells with the ability of precisely regulating their granular enzymes, releasing immunomodulatory mediators and interacting with various immunity players, hence contributing to the resolution of different inflammatory disorders^5–9^.

Amelioration on the stages of fibrosis, hepatic inflammation and steatosis have emerged as central endpoints in therapeutic trials as these are key predictors of clinical outcomes in these patients. Interestingly, these endpoints have been shown to spontaneously improve in patients on the placebo arm of randomized clinical trials at rates reaching 25% of patients in this group^10^. While factors explaining the spontaneous improvement such as weight loss have been identified, in many instances no other specific factors could be found^10^, leading to the concept of a highly active spontaneous resolution process related to the natural evolution of the disease. In this scenario, neutrophils were seen not to recede, but instead have an active role promoting the resolution process^8,11,12^ through mechanisms that are still unclear. Assessing neutrophil roles in the pathophysiology of NASH during the active inflammatory phase as well as the resolution phase of the disease is therefore crucial and possesses important translational implications.

In this study, a murine NASH model was developed through the administration of a high fat-fructose-cholesterol (FFC) diet. This model resembles human NASH the closest by inducing a chronic lipotoxic background leading to release of danger-activated molecular patterns (DAMPs) from injured hepatocytes along with gut dysbiosis and altered intestinal barrier resulting in a strong activation of the innate immunity^13,14^. In this study, by performing a depletion of this population, we investigated the contribution of neutrophils in NASH progression as well as their role in inflammation resolution. Changes in fibrosis levels, inflammation markers and cell infiltration were analyzed as well as differences in the development of resolution promoting mechanisms.

## Materials and methods

### Mice

Male WT C57BL/6J mice aged 8 weeks were purchased from the Jackson Laboratories and housed in a temperature and light cycle-controlled room. All researchers involved in animal experiments complied with relevant animal-use guidelines and ethical regulations during this study. Experimental protocol was approved by the Institutional Animal Care and Use Committee at the University of California San Diego. All efforts were made to minimize pain and distress during experimental interventions. The present study is reported in accordance with the ARRIVE guidelines.

### FFC diet-induced fibrotic NASH and neutrophil depletion treatment

Eight-week old mice were started on a high-saturated fat/cholesterol diet (AIN-76 Western Diet, Test Diet, St. Louis, MO, USA) and water was supplemented with sucrose/fructose (42 g/L) for 17 weeks. The combination of this diet with the sucrose/fructose water was termed FFC diet. In order to study the role of neutrophils during ongoing inflammation, a group of these mice underwent intraperitoneal injections with either anti-Ly6G antibodies (Clone 1A8, Cat #127649, BioLegend, FFC + anti-Ly6G, n = 6), non-specific IgG antibodies (Clone RTK2758, Cat #400565, BioLegend, FFC + IgG, n = 6) as provided by the manufacturer, or equal volume of PBS (FFC + PBS, n = 4) during the last 2 weeks of FFC diet. The administration of the injections was performed in a concentration of 200 μg of antibody per gram of body weight every 4 days until reaching day 14 of treatment, when they were euthanized for blood collection and livers were harvested for analysis, following the protocol of Calvente et al.^8^. A separate group of mice was kept on CD (n = 6) for 17 weeks to serve as the non-injured control.

For the study of the role of neutrophils during NASH resolution, a separate group of mice received a reversal from FFC diet to Control Diet (AIN-76A Control 20% Fat, Test Diet, St. Lois, MO, USA) at week 17 of the regimen in order to allow the start of spontaneous inflammation resolution. CD was administered for 2 weeks either alone (FFC-CD, n = 4) or simultaneously with the administration of intraperitoneal anti-Ly6G antibodies (FFC-CD + anti-Ly6G, n = 3) or non-specific IgG antibodies (FFC-CD + IgG, n = 3) at the same frequency and dosage as described above. Mice were euthanized for blood collection and livers were harvested for analysis. A separate group of mice was kept on the FFC (n = 4) or control diet (n = 4) for 19 weeks to serve as the positive and non-injured CD groups, respectively.

### Liver and serum sample preparation

Mice were sacrificed at the respective terminal end point of diet with or without treatment (2 weeks after starting with the anti-Ly6G antibodies, IgG, PBS, or an equivalent period of time of CD/FFC feeding). Mice were euthanized by being placed in a chamber with a controlled carbon dioxide filling rate (50% of the chamber volume per minute) followed by cervical dislocation. Liver tissue was harvested and distributed in the following manner: two representative sections were immediately fixed in 10% formalin for 24 h and embedded in paraffin; a representative portion was embedded in O.C.T for future frozen sections; samples of 50 μg were placed in 0.5 mL of RNAlater Solution (Lifetechnologies, Carlsbad, CA, USA) for future RNA isolation, and remaining liver tissue was quickly frozen in liquid nitrogen and stored at − 80 °C.

Blood samples were extracted by performing cardiac puncture, and coagulated at room temperature for 30 min. Samples were centrifuged at 300 rpm for 7 min, after which serum was extracted and immediately stored at − 80 °C.

### Histopathology and immunohistochemistry

Liver specimens were fixed in 10% formaldehyde and 70% ethanol for 24 h each and embedded in paraffin. Deparaffinized and rehydrated 5 µm thickness sections were stained with hematoxylin (Sigma-Aldrich, St. Louis, MO, USA) and eosin (Richard Allan, Kalamazoo, MI, USA) for steatosis evaluation. To identify collagen accumulation, sections were incubated for 1 h at RT with an aqueous solution of saturated picric acid (Sigma-Aldrich) mixed with 0.1% Fast Green fetal calf serum (FCS) and 0.1% Direct Red Dye (Sigma-Aldrich). Five randomly selected fields (10× magnification) were photographed and the percentage of Sirius Red-stained area was measured by ImageJ software with an adjusted unchanged threshold.

For immunohistochemical staining, 5 µm thickness sections were deparaffinized and rehydrated with xylene, decreasing ethanol concentrations and distilled water. Sections were incubated for 5 min in 3% hydrogen peroxide for internal peroxidase blockade.

For α-SMA and F4/80 detection, antigen retrieval was performed by incubating the sections in TBS-T with 2% BSA + 1% Triton X-100 for 30 min. For the remaining, this step was performed by incubating the sections in preheated Citrate Buffer pH 6.0 (Dako Reagents) in a 95 °C bath for 20 min. Sections were incubated with rabbit anti-α-SMA (ab124964, 1:500, Abcam), anti-CD11b (ab128797, 1:350, Abcam), anti-CD163 (ab182422, 1:500, Abcam), rat anti-Ly6G (Cat 14-5931-82, 1:200, Invitrogen), anti-Ly6C (ab15627, 1:200, Abcam), or anti-F4/80 (Cat 123106, 1:100, BioLegend) primary antibodies diluted in Dako Antibody Diluent (Odense, Denmark). After incubation with the primary antibodies for 16 h, sections were washed with TBS-T and incubated with ready-to-use HRP-linked anti-rat or rabbit secondary immunoglobulin G antibody (Immpress HRP reagents; Vector Labs, Burlingame, CA) for 1 h at RT. Color was developed with DAB solution (Vector Labs) and nuclei was counterstained with Mayer’s hematoxylin for 2 min, followed by dehydration with increasing ethanol concentrations. Stainings were quantified in 5 randomly selected fields (10× magnification) imaged with a Nanozoomer 2.0HT slide Scanner microscope (Hamamatsu Photonics K.K., Hamamatsu, Japan). The total stained area was analyzed by selecting brown areas using an unchanged threshold value in the macro function of ImageJ (NIH, Bethesda, MD, USA). Results were represented as the average of the percentage of total area occupied by positive cells per field in each specimen.

### Liver function tests

Blood samples were collected during mice sacrifice and serum values of alanine aminotransferase (ALT) were measured according to manufacturer’s instructions (Infinity™ ALT, Thermo Scientific, Waltham, MA, USA).

### Hepatic hydroxyproline quantification

100 mg of liver was homogenized and hydrolyzed by being baked at 110 °C for 18 h with 6 N HCl. Homogenates were filtered and aliquots were evaporated at 60° for 40 min. Crystals were resuspended with 50 μL of water and incubated for 20 min with 100 μL Chloramine-T solution at room temperature. 100 μL of Ehrich’s reagent was then added and samples were incubated at 65 °C for 20 min. Absorbance was measured at 558 nm. Standard seriated curve was made using stock trans-4-hydroxy-l-proline (Sigma, Cat#7279).

### Real-time PCR

Liver tissue underwent RNA extraction with TRIzol Reagent (Sigma-Aldrich) and 50 µg of purified RNA was reverse-transcribed into cDNA by using the qScript cDNA Synthesis Kit (Quantabio). The following gene primer sequences (Table 1) were used. Target gene expression levels were calculated with normalization to the *gapdh* gene expression levels followed by a comparative cycle threshold Ct method (2^−ΔΔCt^).

**Table 1:** Gene primers used for mRNA expression analysis.

Gene Primers	Identifier (Taqman)	Process associated with gene expression
*Gapdh*	Mm99999915_g1	Housekeeping gene
*Col1a1*	Mm00801666_g1	Fibrosis
*Col3a1*	Mm01254476_m1	Fibrosis
*Timp1*	Mm01341361_m1	Fibrosis
*Chi3l1*	Mm00657889_mH	Fibrosis and inflammation
*Acta2*	Mm00725412_s1	Hepatic stellate cell activation
*Tgfb*	Mm01178820_m1	Hepatic stellate cell activation
*Ctgf*	Mm01192933_g1	Hepatic stellate cell activation
*Ccl2*	Mm00441242_m1	Monocyte chemotaxis
*Ccr2*	Mm99999051_gH	Monocyte chemotaxis
*Nlrp3*	Mm00840904_m1	NLRP3 inflammasome pathway
*Casp1*	Mm00438023_m1	NLRP3 inflammasome pathway
*Il1b*	Mm00434228_m1	NLRP3 inflammasome pathway
*Tnfa*	Mm00443258_m1	Inflammation and resolution
*Il4*	Mm00445259_m1	Inflammation resolution
*Il10*	Mm01288386_m1	Inflammation resolution
*Il6*	Mm00446190_m1	Inflammation resolution
*Mmp2*	Mm00439498_m1	Pro-fibrotic metalloproteinase
*Mmp8*	Mm00439509_m1	Tissue repair and remodeling
*Mmp9*	Mm00442991_m1	Tissue repair and remodeling
*Mmp10*	Mm00444630_m1	Tissue repair and remodeling

### Immunoblot analysis

Protein fraction was purified from approximately 100 mg of liver biopsy using RIPA lysis buffer for 40 min on ice. 25 μg of total tissue protein were loaded in SDS with 3% mercaptoethanol, heated for 5 min at 95 °C, electrophoresed via an Any kDa precast Tris–Glycine (Bio-Rad) at 80 V for 30 min, followed by 120 V until the end of the run. Tris–glycine-SDS was used as running buffer. Proteins were transferred from the gel into a nitrocellulose membrane in a Trans Blot Turbo Transfer system (Bio-Rad) for 7 min at 25 V. An ethanol based solution was used as transfer buffer (Bio-Rad), followed by blockage of the membrane for 1 h with the Intercept Blocking Buffer (Li-Cor) at RT.

Membranes were then incubated with mouse anti caspase-1 (Ag 20B-0044-C100, AdipoGen, 1 mg/mL), anti-α-tubulin (T6199, Sigma-Aldrich, 1/5000), anti-MMP2 (ab92536, Abcam, 1:1000), rabbit anti-IL1β (ab9722, Abcam, 0.2 mg/mL), anti –α-SMA (ab124964, Abcam, 1:1000) or goat anti-arginase 2 (ab60176-100, Abcam, 0.03 mg/mL) primary antibodies at 4 °C overnight. Membrane was washed two times in TBS-tween and incubated with HRP-linked anti-mouse, rabbit or goat IgG antibodies (Li-Cor) for 1 h at RT. Protein bands were scanned with the Odissey Infrared Imager (Li-Cor) and analyzed with the Image Studio software.

### Statistical analysis

Analyses were performed with GraphPad (version 8.4.2; GraphPad Software Inc., La Jolla, CA, USA). Data are shown as means ± SEM. Data from multiple groups were compared with 1-way analysis of variance (ANOVA) followed by the Turkey post hoc test. p < 0.05 was considered a significant difference.

## Results

### Neutrophil depletion in mice fed with FFC diet for 17 weeks induced a significant improvement of hepatic inflammation and fibrosis

**Figure 1:**
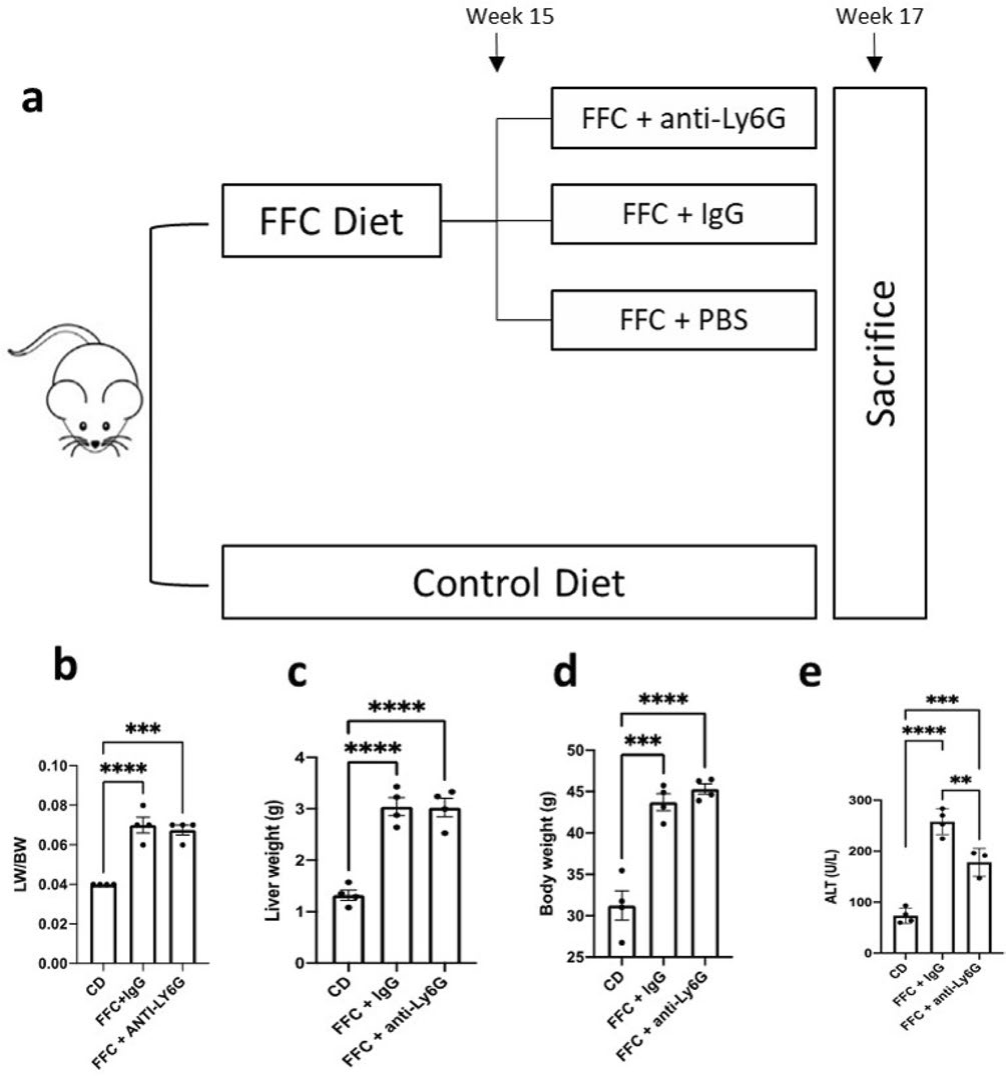
Neutrophil depletion treatment after 15 weeks of high fat-fructose-cholesterol diet (FFC). (**a**) Mice were fed with FFC diet for 15 weeks and were administered with IgG (FFC + IgG, n = 6) or anti-Ly6G (FFC + anti-Ly6G, n = 6) intraperitoneal injections for 14 days until sacrifice. Control diet was used as a NASH negative control (CD, n = 6). Liver/body weight ratio (**b**), liver weight (**c**), body weight (**d**) and serum alanine transaminase (**e**) were compared between the mentioned groups with 1-way analysis of variance (ANOVA) followed by the Turkey post hoc test. P < .05 was considered a significant difference. *Ly6G* lymphocyte antigen 6 complex locus G6D, *LW/BW* liver/body weight ratio, *ALT* alanine aminotransferase.

Male C57BL/6J were fed with FFC diet or CD for a total of 17 weeks. During the last 2 weeks of diet, the FFC receiving mice were administered with intraperitoneal injections of anti-Ly6G antibodies for neutrophil depletion, IgG antibodies, or PBS as controls (Fig. 1a). While FFC diet-receiving mice showed a LW/BW ratio increase of approximately 75% when compared to the CD group, no significant difference was observed among the different treatments on FFC (Fig. 1b). The same trend was observed when comparing liver and body weights separately (Fig. 1c,d). When hepatocellular damage was assessed via serum ALT levels, a significant decrease was seen in the neutrophil depleted mice when compared to the FFC + IgG group (178 ± 16 vs. 258 ± 13; *p* = 0.005) (Fig. 1e).

**Figure 2:**
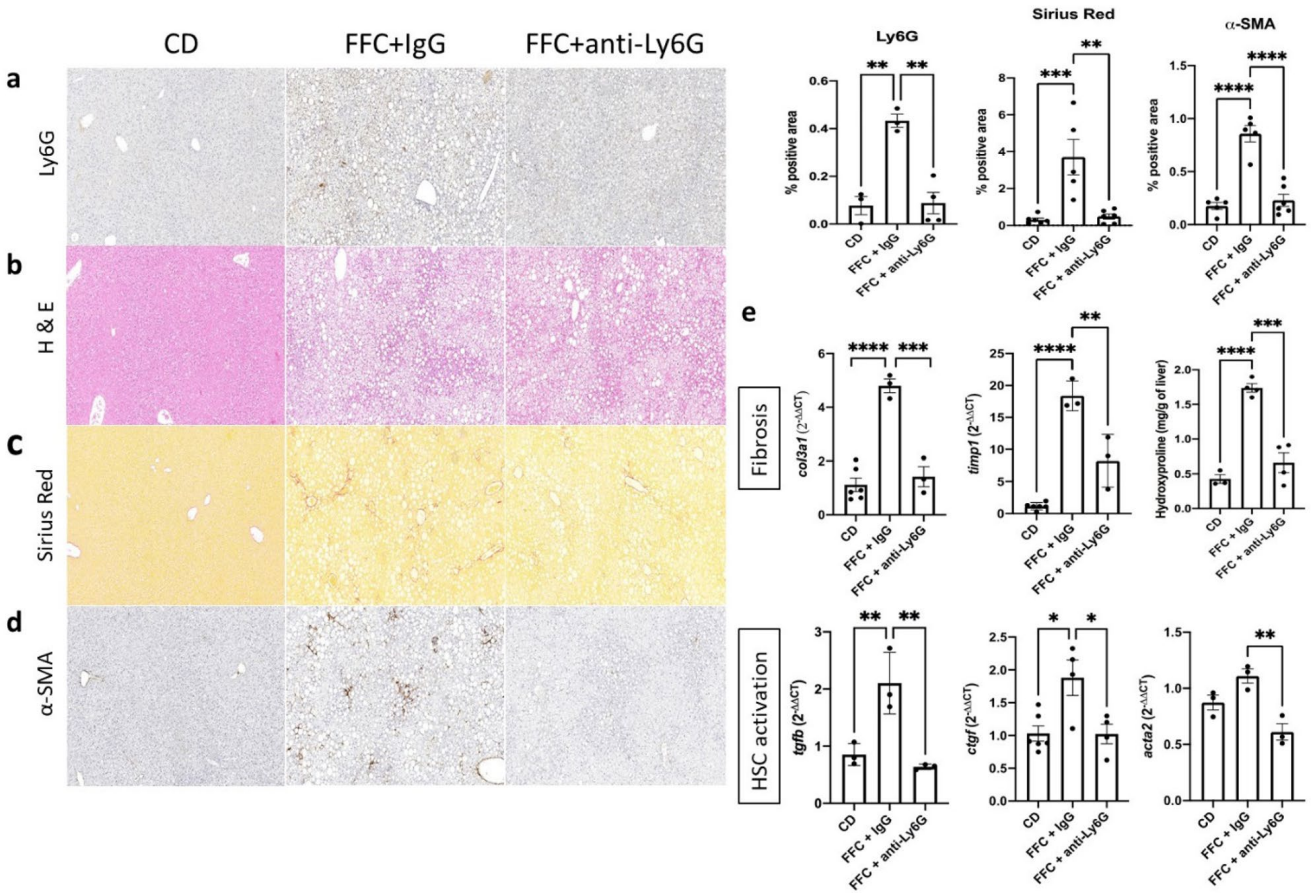
Hepatic steatosis, fibrosis and hepatic stellate cell (HSC) activation are ameliorated in neutrophil depleted mice during NASH development. Neutrophil marker Ly6G (**a**), steatosis (**b**) and fibrosis (**c**) levels were assessed via IHC, H&E and Picrosirius Red stainings IHC respectively on paraffin-embedded tissue sections. Activated α-SMA^+^ HSCs (**d**) were detected via IHC. Quantification and ANOVA comparison were made between the control diet and IgG or anti-Ly6G regimen receiving groups during ongoing NASH development. (**e**) mRNA expression of hepatic fibrosis and HSC activation related genes and hydroxyproline levels were analyzed between the mentioned groups and compared with ANOVA IHC. *CD* control diet, *α-SMA/acta2* alpha smooth muscle actin, *IHC* immunohistochemistry, *col3a1* collagen type 3 alpha 1 chain, *timp1* tissue inhibitor of metalloproteinases 1, *tgfb* transforming growth factor beta, *ctgf* connective tissue growth factor.

Hepatic inflammation and fibrosis were assessed through H&E and Picrosirius Red (PSR) stainings respectively throughout the groups (Fig. 2a–c). As the assessment of FFC + PBS and FFC + IgG control groups showed highly similar results throughout the analysis of this study, hence excluding the IgG isotype as a potential effector of any changes observed in the neutrophil-depleted group, only the latter group was included in the data representations for summarizing purposes. All FFC diet-fed mice showed a substantial increase in fat deposition in the form of micro and macrovesicular steatosis compared to the CD-receiving group and the changes were similar when comparing the FFC, FFC + IgG and FFC + anti-Ly6G groups (Fig. 2b). In contrast, PSR showed a significant decrease in collagen deposition in the FFC + Ly6G group when compared to the FFC + IgG group (0.49 ± 0.14 vs 3.71 ± 0.96; *p* = 0.0013), demonstrating that neutrophil depletion induced the reduction of collagen deposition (Fig. 2c). Hydroxyproline concentrations were also compared and showed significantly lower levels in the neutrophil depleted group (0.66 ± 0.14 vs 1.738 ± 0.06; *p* = 0.0001) (Fig. 2e). Consistent with these results, neutrophil depletion also lead to a reduction of gene expression levels related to liver fibrosis including collagen type 3 (Col3a1: 1.4 ± 0.37 vs. 4.8 ± 0.25; *p* = 0.0002) and tissue inhibitor of metalloproteinase-1 (Timp-1) (8.2 ± 2.4 vs. 18 ± 1.3; *p* = 0.001) (Fig. 2e).

### Neutrophil depleted mice on continuous FFC diet show amelioration of HSC activation

Infiltration of the liver with monocyte-derived macrophages and other immune cells in NASH promote the production of various pro-fibrogenic and pro-inflammatory cytokines^15^ that contribute to the activation and trans-differentiation of HSC into myofibroblast-like cells with contractile properties and increased proliferation rates. Activated alpha smooth muscle actin (α-SMA)-positive hepatic stellate cells (HSC) compose the main source of collagen and extracellular matrix (ECM) production in NASH, leading to parenchymal destruction due to vascular distortion and structural alteration of the organ architecture^16^. As fibrosis was shown to be substantially decreased in the FFC + anti-Ly6G group we proceeded analyzing the activation of HSCs through anti-α-SMA IHC (Fig. 2d) and related genes RNA expression profile. Results showed a significant decrease of α-SMA^+^ cells (0.23 ± 0.14 vs. 0.86 ± 0.17; *p* < 0.0001) as well as lower levels of HSC activation related signal pathways such as transforming growth factor beta (*tgfb)* and connective tissue growth factor (*ctgf)* in the neutrophil depleted group (3.18 and 1.8-fold decreases respectively; *p* = 0.032 and 0.009) (Fig. 2e). This confirms neutrophils are highly involved in the activation of HSCs, resulting in the increased fibrogenesis during NASH development.

### Macrophage population assessment showed decreased CCR2 mediated cell infiltration and lower levels of inflammatory macrophage activity in the neutrophil depleted group

**Figure 3:**
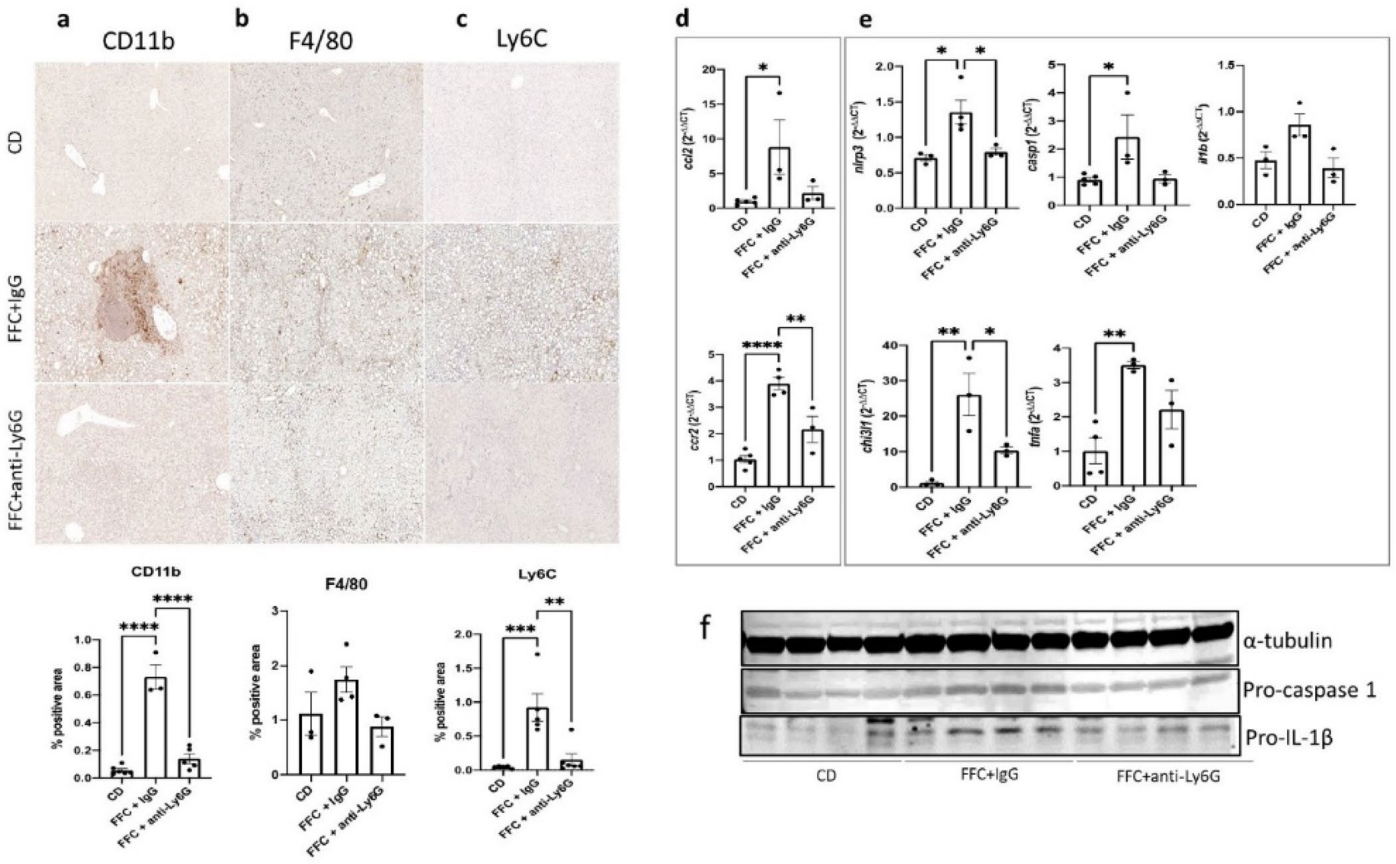
Cellular infiltration and pro-inflammatory macrophage activity are abrogated in neutrophil depleted mice during NASH development. IHC for detection of monocyte derived macrophages and other CD11b^+^ infiltrating myeloid cells (**a**), hepatic resident F4/80^+^ Kupffer cells (**b**) and pro-inflammatory Ly6C^hi^ monocytes/macrophages was performed and quantified for comparison among the control diet receiving group and the mice receiving either IgG or anti-Ly6G antibodies during ongoing FFC diet . mRNA expression was analyzed via RT-qPCR and fold-change was compared between the mentioned groups regarding the main monocyte chemotactic pathway CCL2/CCR2 (**d**) and pro-inflammatory macrophage activity markers such as components of the NLRP3 inflammasome pathway, chitinase-3 like 1 protein (CHI3L1) and TNF-α (**e**). Data was compared by using ANOVA analysis followed by the Turkey post hoc test. Western Blots targeting components of the NLRP3 downstream cascade were performed to compare the mentioned groups in a protein expression level (**f**). α-tubulin (band imported from Supplementary Fig. 1b) was used as housekeeping protein in both assessments. Full-length blottings of pro-caspase 1 and pro-IL-1β are shown in Supplementary Fig. 1b and 2, respectively. *Ccl2* C–C motif chemokine ligand 2, *ccr2* C–C motif chemokine receptor 2, *NLRP3* NLR family pyrin domain containing 3, *CHI3L1* chitinase 3-like 1, *TNF-α/tnfa* tumor necrosis factor alpha.

Infiltration and communication amongst inflammatory cells in the liver are main hallmarks of NASH. Anti-CD11b IHC in the liver tissues of the various groups was performed to detect monocyte derived macrophages and other infiltrating myeloid cells from the bloodstream and showed less concentration of CD11b^+^ cells in the FFC + anti-Ly6G group compared to the IgG treated group (0.14 ± 0.04 vs. 0.73 ± 0.09; *p* < 0.0001) (Fig. 3a). As C–C motif chemokine receptor 2 (CCR2)/C–C motif chemokine ligand 2 (CCL2) compose the main chemotactic pathway for bloodstream monocytes that will differentiate into pro-inflammatory hepatic macrophages, RNA expression was measured, showing a significant decrease of *ccr2* levels (2.2 ± 0.5 vs. 3.9 ± 0.23; *p* = 0.005) and down trending levels of *ccl2* in the neutrophil depleted mice (Fig. 3d). In agreement with these findings, while total liver F4/80^+^ Kupffer cells population did not show significant differences between the FFC diet receiving groups, a significant decrease of pro-inflammatory Ly6C^hi^ monocytes and macrophages were observed in liver sections of the FFC + anti-Ly6G group compared to the FFC + IgG group (0.15 ± 0.09 vs. 0.92 ± 0.2; *p* = 0.0017) (Fig. 3b,c).

Signal pathways closely related to the activity of proinflammatory Ly6C^hi^ macrophages were measured at both gene and protein expression levels and reflected a significant amelioration in the neutrophil depletion treatment group (Fig. 3e,f). The nucleotide-binding oligomerization domain-like receptor protein 3 (NLRP3) pathway, described to be active in pro-inflammatory myeloid cells and crucial for the progression into NASH^17,18^, showed reduced RNA expression levels of both the inflammasome coding gene (1.7-fold decrease; *p* = 0.045) and down trending expression of downstream pro-caspase 1 and pro-IL-1β in the FFC + anti-Ly6G group (Fig. 3e,f). Neutrophil depleted mice also showed a significantly decreased level of chitinase-3-like 1 protein (CHI3L1) expression (2.6-fold decrease; *p* = 0.044) (Fig. 3e), a fibrosis marker described in NASH and other inflammation-related disorders^19^, opening the question of whether neutrophils are a major source of this protein or an important inducer of its upregulation. Therefore, neutrophils are crucial for inflammatory infiltration, monocyte differentiation into Ly6C^hi^ pro-inflammatory macrophages and induction of signaling pathways involved in NASH progression.

### Diet reversal into low fat control diet after 17 weeks of FFC leads to spontaneous resolution of liver inflammation

**Figure 4:**
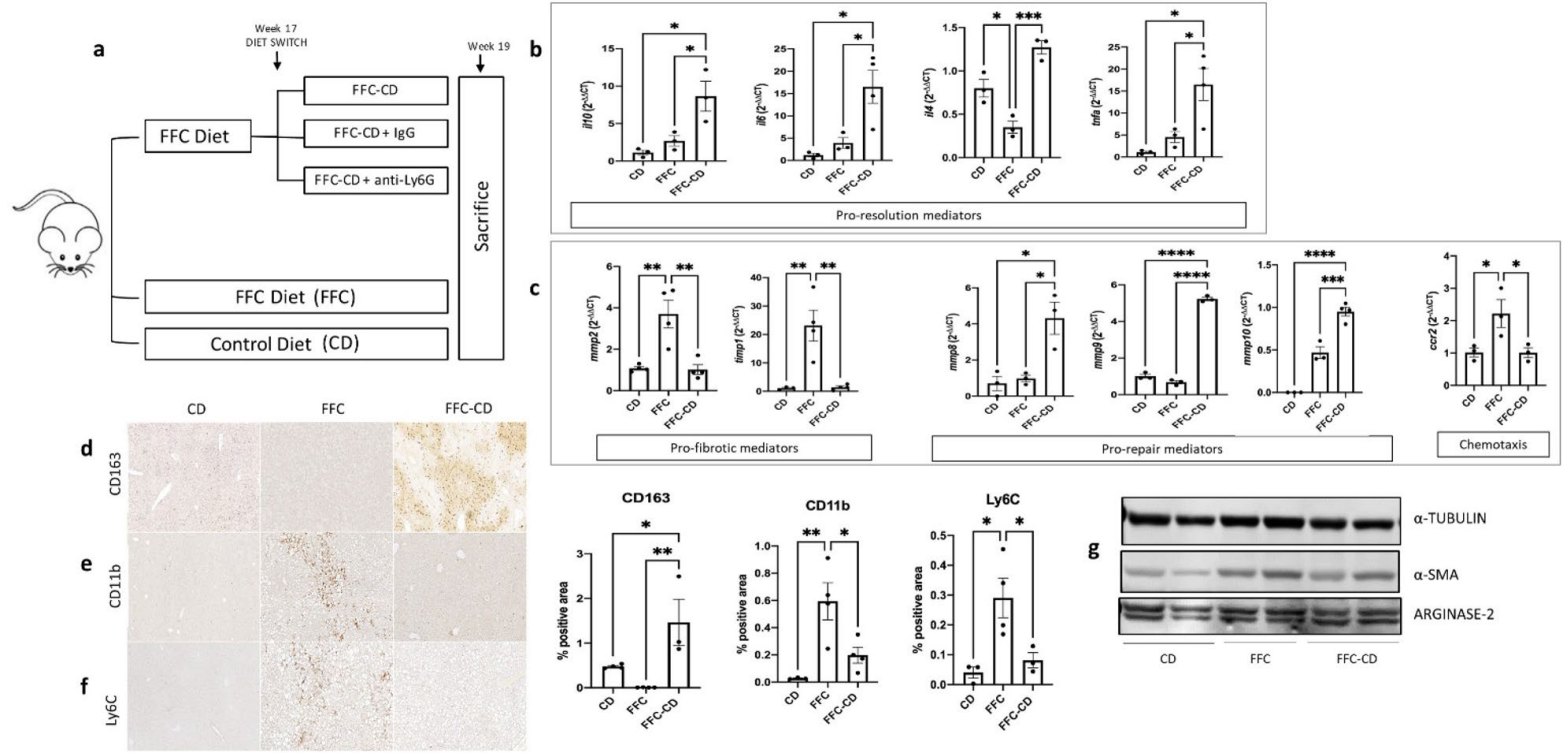
NASH inflammation resolution induced by a diet reversal from high fat diet to control diet. (**a**) After 17 weeks of high fat-fructose-cholesterol diet (FFC), mice underwent a 2-week diet reversal into control diet (CD), allowing spontaneous resolution of inflammation. During the diet reversal period, mice received treatment with anti-Ly6G (FFC-CD + anti-Ly6G, n = 3), IgG (FFC-CD + IgG, n = 3) or no treatment (FFC-CD, n = 4). Groups receiving 19 weeks of FFC (n = 4) or CD (n = 4) were used as positive and negative NASH controls respectively. mRNA expression was measured via RT-qPCR and fold change was analyzed between control diet, FFC and diet reversal receiving groups through ANOVA comparison: expression of mediators with pro-resolutive roles (**b**), fibrotic mediators (mmp2, timp1) vs. pro-repairing matrix metalloproteinases (mmp8, mmp9 and mmp10) and chemotactic marker ccr2 were compared (**c**) among the mentioned groups. Detection of pro-resolution profile CD163^+^ macrophages (**d**), monocyte-derived macrophages and other infiltrating CD11b^+^myeloid cells (**e**) and pro-inflammatory Ly6C^hi^ monocytes/macrophages (**f**) was performed via IHC and quantification. (**g**) Western blot was used to assess differences in protein expression of hepatic stellate cell activation marker α-SMA (cropped from Supplementary Fig. 3a) and pro-resolutive M2 macrophage marker arginase-2 (cropped from Supplementary Fig. 3b) between the mentioned groups. α-tubulin expression (band imported from Supplementary Fig. 3a) was used as housekeeping control. *Mmp* matrix metalloproteinases.

Spontaneous resolution of liver inflammation was achieved by reversing FFC diet at week 17 into CD (FFC-CD) for 14 days until sacrifice. A separate group received either FFC diet with no diet reversal (FFC) or CD for 19 weeks in order to serve as NASH positive control and no injury negative controls respectively (Fig. 4a).

**Figure 5:**
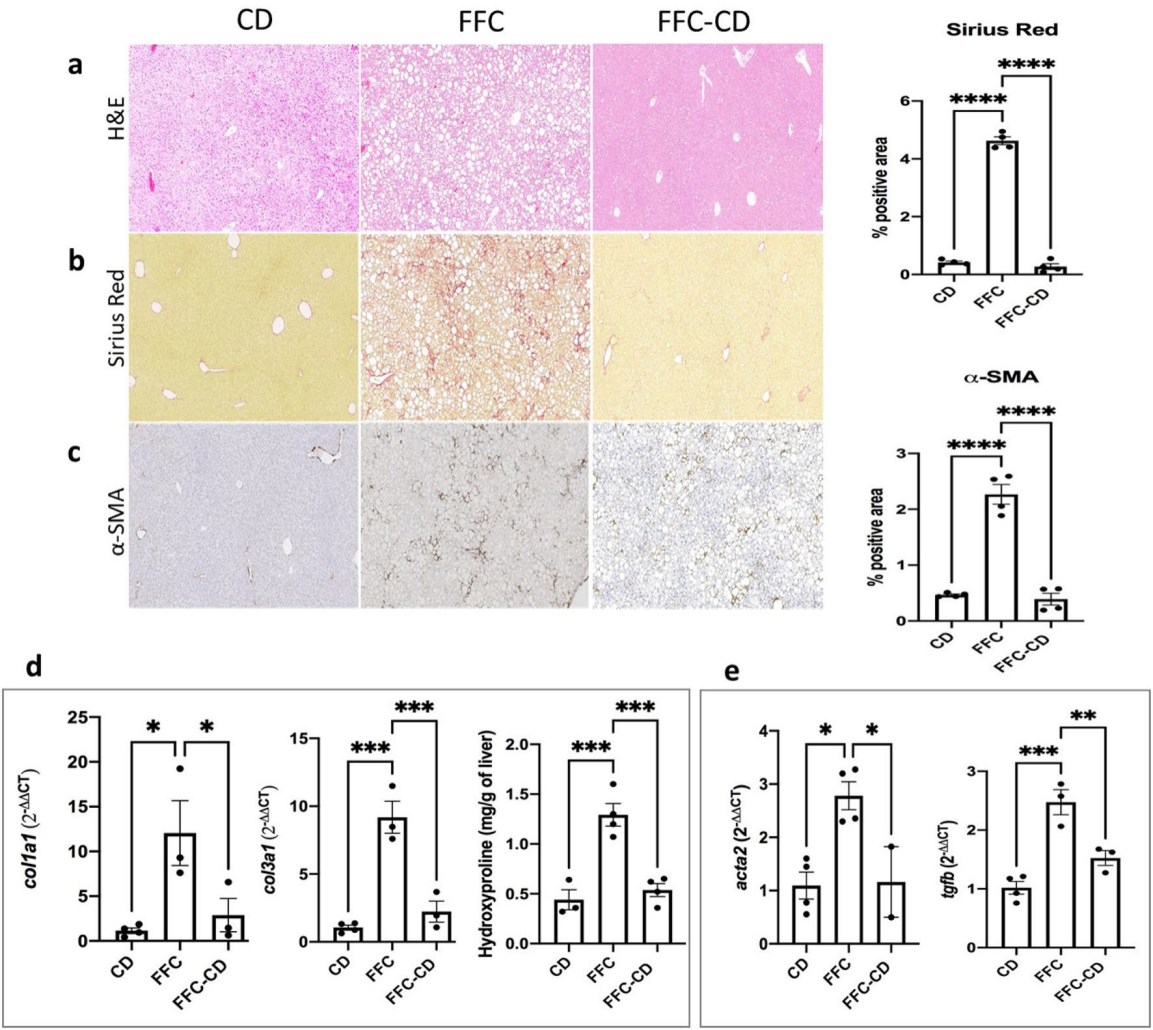
Hepatic steatosis, fibrosis and hepatic stellate cell (HSC) activation are ameliorated in NASH resolution induced by diet reversal. Steatosis (**a**), fibrosis (**b**) and activated HSC activation (**c**) were detected via hematoxylin, Sirius Red and α-SMA IHC stainings respectively in paraffin embedded tissue sections. Hydroxyproline levels and mRNA expression of fibrosis-related genes (**d**) and HSC activation markers (**e**) were analyzed through RT-qPCR among the control diet, FFC and diet reversal receiving groups. Data was analyzed with ANOVA comparison followed by the Turkey post hoc test.

Essential factors for spontaneous resolution of liver inflammation include a decrease of the pro-inflammatory/anti-inflammatory cytokines ratio, a phenotypic switch turning hepatic macrophages into a pro-restorative profile, and release of pro-reparative and remodeling factors such as specific matrix metalloproteinases (MMPs). Expression of cytokines with pro-resolutive roles such as TNF-α^20,21^, IL-6^22^, IL-10 and IL-4^23^ were significantly increased (3.56, 4.1, 3.18, 3.7-fold increases respectively; *p* = 0.036, 0.03, 0.03, 0.0006) in the diet change group when compared to the FFC group (Fig. 4b). Markers of pro-restorative profile macrophages such as CD163 (IHC 1.5 ± 0.52 vs. 0.01 ± 0.001; *p* = 0.012) (Fig. 4d) and arginase-2 (Fig. 4g) were observed to be significantly increased in contrast to decreased infiltrating CD11b^+^ cells (0.2 ± 0.058 vs. 0.59 ± 0.14; *p* = 0.037) (Fig. 4e) and *ccr2* expression (2.2-fold decrease; *p* = 0.047) (Fig. 4c) within the FFC-CD group. Differentiation into pro-inflammatory Ly6C^hi^ monocytes/macrophages was also evidenced to be lowered (IHC 0.082 ± 0.03 vs. 0.29 ± 0.07; *p* = 0.046) (Fig. 4f). These findings reveal a successful phenotypic switch in macrophages when inflammation resolution takes place. Important repair and remodeling mediators such as collagen degrading matrix metalloproteinases (MMPs) are released by pro-restorative macrophages, leading to collagen degradation and parenchymal recovery^24,25^. Expression of pro-fibrotic MMP-2 and tissue inhibitor of metalloproteinases 1 (TIMP-1) was significantly lowered (2.64 and 16.8-fold decreases in *mmp2* and *timp1* mRNA; *p* = 0.034 and 0.045) while anti-fibrotic MMP-8, 9 and 10 were seen to be increased in the FFC-CD group when compared to the FFC group (4.43, 7.8, 2.02-fold increases respectively; *p* = 0.015, < 0.0001, 0.0007) (Fig. 4). In fact, while changes in steatosis were inconclusive, PSR stainings and hydroxyproline levels showed a significant decrease of fibrosis levels (0.27 ± 0.1 vs. 4.7 ± 0.35; *p* < 0.0001 and 0.54 ± 0.07 vs. 1.29 ± 0.1; *p* < 0.001) (Fig. 5a,b,d)*.* Additionally, RNA expression of collagen coding genes were significantly lowered (4.18 and 4.11-fold decreases in *col1a1* and *col3a1* respectively*; p* = 0.049 and 0.001) (Fig. 5d). α-SMA^+^ myofibroblasts were comparably scarce (IHC 0.39 ± 0.1 vs. 2.4 ± 0.13; *p* < 0.0001) (Fig. 5c) and HSC activating genes were significantly less expressed in the diet reversal group when compared to the FFC group (2.4 and 1.6-fold decrease in *acta2* and *tgfb* respectively; *p* = 0.039 and 0.009) (Fig. 5e).

### Neutrophil depletion treatment in association with DC delays SRLI and aggravates hepatic inflammation

**Figure 6:**
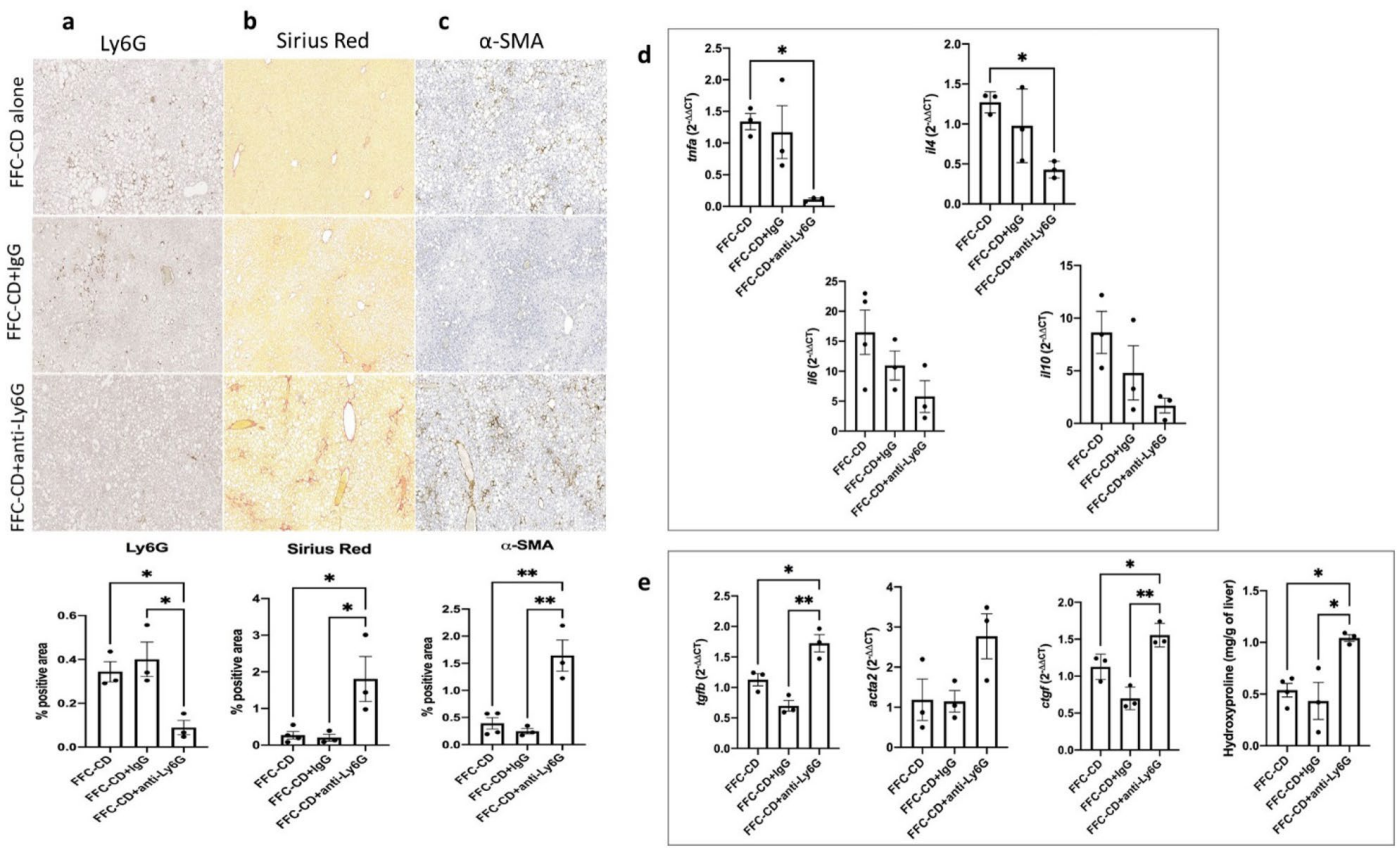
Fibrosis and hepatic stellate cell (HSC) activation are exacerbated in neutrophil depleted mice during inflammation resolution. Neutrophil depletion was achieved via administration of anti-Ly6G antibodies during the 2-week diet reversal-induced NASH inflammation resolution. Ly6G^+^ neutrophils (**a**), fibrosis (**b**) and α-SMA^+^ activated HSCs (**c**) were detected through paraffin-embedded tissue sections stainings and analyzed by ANOVA comparison between mice undergoing diet reversal alone (FFC-CD), and diet reversal in association with either IgG (FFC-DC + IgG) or anti-Ly6G antibodies (FFC-CD + anti-Ly6G) administration. mRNA expression of genes associated with inflammation resolution and HSC activation was assessed comparing fold-changes between the mentioned groups. Hydroxyproline levels were also compared among the mentioned groups by ANOVA followed by the Turkey post hoc test (**d**,**e**).

As reversing the diet into CD was shown to be effective on allowing inflammation resolution, neutrophil role in this process was assessed by administrating anti-Ly6G (FFC-CD + anti-Ly6G) or IgG (FFC-CD + IgG) injections in parallel to the diet reversal phase (Figs. 4a, 6a).

**Figure 7:**
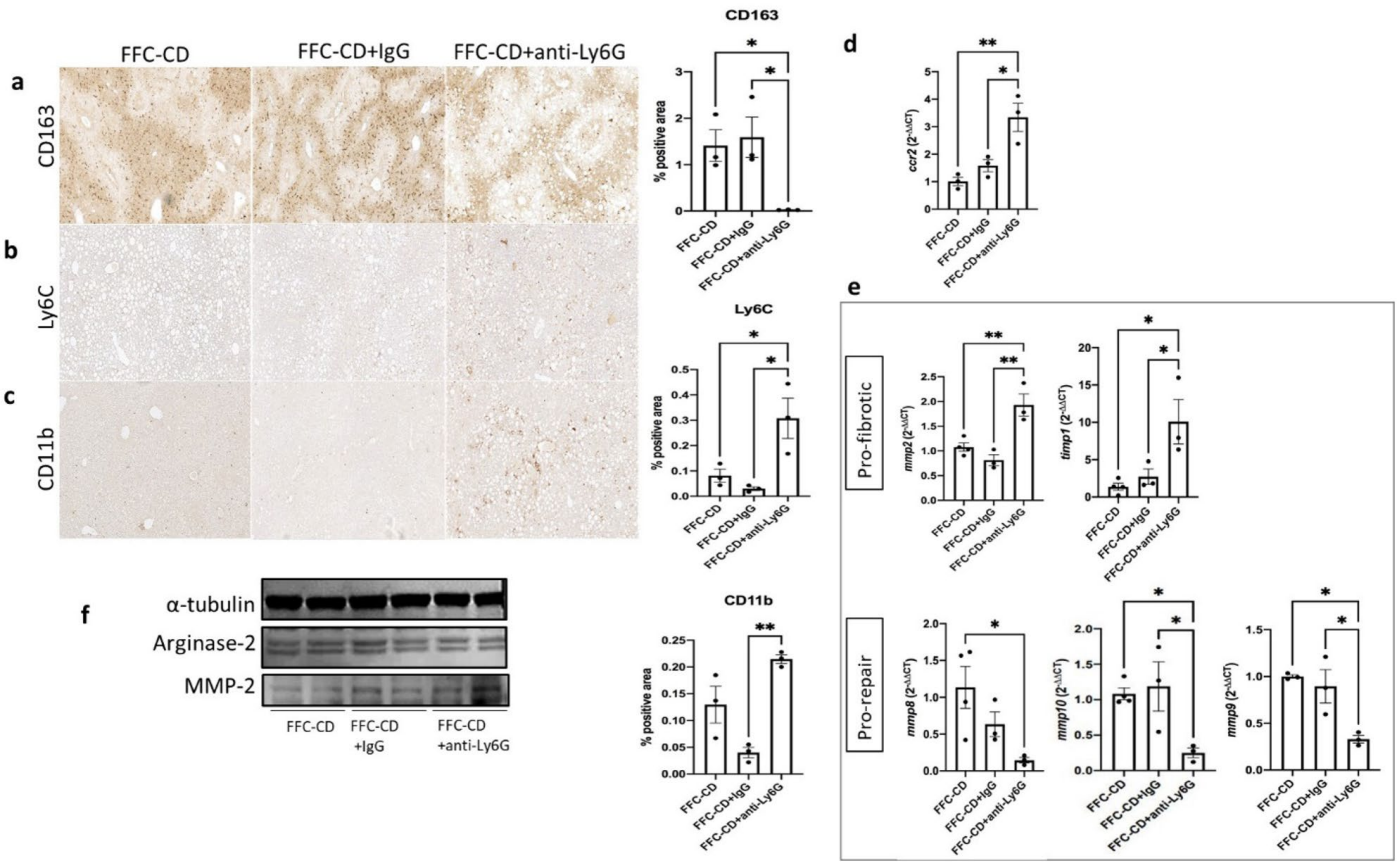
Phenotypic switch into pro-resolution macrophage population and release of pro-repairing mediators are impaired in neutrophil depleted mice during inflammation resolution. Detection of CD163^+^ pro-restorative macrophages (**a**), pro-inflammatory Ly6C^hi^ monocytes/macrophages (**b**) and infiltrating CD11b^+^ myeloid cells (**c**) were detected via IHC and quantified for ANOVA comparison between the groups receiving diet reversal alone (FFC-CD), and diet reversal in association with either IgG (FFC-CD + IgG) or anti-Ly6G (FFC-CD + anti-Ly6G). mRNA expression was compared and expressed as fold changes. Monocyte chemoattractant receptor ccr2 (**d**) and changes in the expression of pro- (mmp2, timp1) versus anti-fibrotic (mmp8, mmp9, mmp10) mediators were compared among the mentioned groups (**e**). (**f**) Western blots for protein expression assessment was performed targeting pro-restorative macrophage marker arginase 2 (cropped from Supplementary Fig. 4) and pro-fibrotic MMP-2 (cropped from Supplementary Fig. 5b). α-tubulin expression (band imported from Supplementary Fig. 4) was illustrated as housekeeping control.

Results showed an important impairment of the mechanisms leading to inflammation resolution. Cytokines involved in inflammation resolution TNF-α and IL-4 were significantly decreased when compared to the FFC-CD group (11.96 and 2.96 fold decrease respectively; *p* = 0.032, 0.03). IL-10 and IL-6, while not achieving statistical significance, still showed a considerable down-trending expression in the neutrophil depleted group (Fig. 6d). PSR and α-SMA IHC showed increased levels of fibrosis (1.8 ± 0.62 vs. 0.2 ± 0.094; *p* = 0.03) and HSC activation (1.6 ± 0.29 vs. 0.25 ± 0.05; *p* = 0.002) respectively in the FFC-CD + anti-Ly6G group when compared to the non-depleted groups (Fig. 6b,c). Consistently, hydroxyproline levels were significantly higher in the neutrophil depleted group (1.043 ± 0.03 vs. 0.43 ± 0.18; *p* = 0.013), as well as the expression of HSC activation-related genes (1.5 and 1.38-fold increase in *tgf* and *ctgf* mRNA respectively; *p* = 0.002 and 0.04) (Fig. 6e). When assessing the macrophage subpopulations, phenotypic switch into a pro-restorative profile was shown to be highly impaired in the FFC-CD + anti-Ly6G group. Pro-restorative CD163^+^ macrophages and its marker arginase 2 were notably scarce in the neutrophil depleted group (IHC 0.024 ± 0.004 vs. 1.6 ± 0.43; *p* = 0.03), while Ly6C^hi^ pro-inflammatory monocytes and macrophages were priming this population (IHC 0.31 ± 0.08 vs. 0.03 ± 0.008; *p* = 0.016) (Fig. 7a,b,f). Inflammatory myeloid cells infiltration was significantly augmented in the FFC-CD + anti-Ly6G group (IHC CD11b 0.21 ± 0.008 vs. 0.04 ± 0.01; *p* = 0.003 and 3.3-fold increase in *ccr2* mRNA; *p* = 0.023) (Fig. 7c,d). As pro-restorative cells had lowered activity levels, MMPs were also expressed in a fibrosis-favoring manner: expression of pro-fibrotic *mmp2* and *timp1* were significantly increased in the DC + Ly6G group (1.79 and 7.3-fold increases respectively; *p* = 0.003 and 0.045), while pro-repair *mmp10* and *mmp8* (4.4 and 7.8-fold decreases respectively; *p* = 0.03 and 0.042) were decreased when compared to the FFC-CD group (Fig. 7e).

In summary, inverse effects were observed when neutrophil depletion was performed alongside inflammation resolution when compared to the effects seen when the same treatment was performed during NASH development. These results support the novel concept that neutrophils are crucial players engaged in the active mechanisms of liver resolution by ameliorating the activation of HSCs and promoting phenotypic switch in macrophages into a pro-restorative profile.

## Discussion

The primary aim of our study was to assess the effects of neutrophil depletion both during the development of NASH in FFC fed mice as well as during the resolution process via diet reversal into CD. FFC diet-induced NASH is a dietary murine model that recapitulates many features of human NASH pathophysiology as it resembles the metabolic syndrome, systemic inflammation and typical liver histopathological changes observed in these patients^26^.

Results demonstrated a protective role of this intervention when performed along with continuous FFC diet feeding. While no differences were observed in liver steatosis or liver/body weight ratios, a significant decrease in fibrosis, HSC activation, cell recruitment and activity of pro-inflammatory Ly6C^hi^ macrophages was observed in the neutrophil depleted group when compared to the non-depleted control groups. These findings lead to the conclusion that neutrophils are key players in the development of hepatic inflammation and fibrosis regardless of hepatic steatosis. An important crosstalk could be proposed involving neutrophils as activators of fibrogenic mechanisms by regulating inflammation effector mechanisms in NASH. These current results support the findings of a recent report by Zhou et al. in a murine model of combined non-alcoholic and alcoholic steatohepatitis induced by the administration of a high fat diet (HFD) and a binge of ethanol where neutrophils were found to be crucial for the activation of HSCs in part by releasing ROS, while these promoted neutrophil survival via the production of GM-CSF and IL-15^27^. Recruitment of inflammatory myeloid cells was also observed to happen in a neutrophil-dependent manner, as anti-Ly6G administration at this time also showed strongly ameliorated chemotaxis and activation of myeloid cells into pro-inflammatory Ly6C^hi^ profile. Nakashima et al. demonstrated the important contribution of activated F4/80^+^ CD11b^+^ macrophages in a high fat-cholesterol diet-induced hepatitis by enhancing TNF-α production and stimulating the TNF/FasL pathway in NKT cells^28^, which could be ameliorated by suppressing the recruitment of these cells by the means of neutrophil depletion. Other soluble factors released by neutrophils such as elastase^29^, lipocalin-2^30^, and ROS^31^ were also found to contribute to the recruitment and activation of inflammatory macrophages and further neutrophils. Neutrophilic oxidative burst, besides also being a strong chemotactic contributor, was also seen to be involved in a robust activation of the p38 pathway in hepatocytes, highly sensitizing them to apoptotic death^31^.

The role of neutrophils during NASH spontaneous resolution remains poorly understood. Inflammation resolution has been traditionally thought as a passive event that gives place once the noxious stimuli cease. But instead, it was found that highly dynamic and active cellular interactions are in charge of counteracting the dysregulated inflammatory response of NASH in order to restore the hepatic function and architecture^11^. Important roles have been attributed to neutrophils as negative regulators of inflammation. During acute inflammation, neutrophils are not only vital for the clearance of debris and pathogens, but also for the recovery of tissue homeostasis^32^. Neutrophil depletion performed in multiple timepoints in a murine model of methacholine deficient (MCD) diet-induced NASH showed beneficial effects on hepatic lipid accumulation and inflammation, but these effects could not be observed after the 8th week of diet. In fact, worsened inflammation after this timepoint suggested that anti-inflammatory mediators such as IL-1Rα released by neutrophils were necessary for stopping inflammation progress and promoting resolution^29^. We previously showed neutrophil release of miR-223 to be crucial for spontaneous resolution of NASH by silencing the NLRP3 inflammasome expression in hepatic macrophages, therefore promoting phenotypic switch into a restorative profile^8^. Potential therapeutic strategies upon the discovery of these mechanisms include the modulation of the miR-223 pathway, either through the application of exogenous synthetic microRNA^33^ or via regulation of upstream promoting pathways such as the myeloid cell specific IL-6 signaling^22^. Neutrophil depletion was also associated with decreased early collagen degradation in resolving cholestatic rat livers due to decreased production of MMP8 in this population as well as lower activation of MMPs that are responsible for successful repair^34^. ROS released by neutrophils have also shown to be essential for macrophage phenotypic switch and hepatocyte regeneration during the resolution phase after an acetaminophen overdose model^12^.

In our study, spontaneous resolution state was established through a 2-week diet reversal into CD after 17 weeks of FFC administration. Evidence of parenchymal restoration was observed by ameliorated fibrosis, along with lowered HSC activation. Recruitment of myeloid cells was significantly diminished, and an efficient phenotypic switch from inflammatory to pro-restorative macrophages was evidenced by IHC and RNA and protein analysis. Repair and remodeling mediators such as metalloproteinases 8, 9, 10 were highly expressed, unlike the pro-fibrotic MMP-2 and TIMP-1, both of which showed an opposite trend. When neutrophil depletion effects were evaluated in this setting, opposite changes were observed: fibrosis and HSC activation levels were significantly exacerbated as well as myeloid cell infiltration. Defective phenotypic switch was evidenced as inflammatory Ly6C^hi^ macrophages were predominating over pro-restorative CD163^+^ cells. Expression of MMPs was also inversed, showing a high expression of pro-fibrotic factors (MMP2, TIMP-1) rather than collagen-degrading pro-repair MMPs.

The dynamical processes that occur throughout NASH development and resolution harden the dissection of neutrophils specific roles. Neutrophil depletion studies have been traditionally driven in the setting of inflammation development, and often, paradoxical results in where these interventions worsen inflammation and fibrosis instead of ameliorating them have suggested the possibility of neutrophils engaging in inflammation resolution^8,29,35^. In the current study, we have shown that neutrophil roles in murine NASH varies depending on the environment within the inflammation-resolution cycle, describing phase-dependent contrasting roles of neutrophils as triggers and pro-resolutive mediators of liver injury and fibrosis associated with diet-induced NASH in mice. It is thus important to contemplate the neutrophil population in a global and parallel manner considering this dual behavior when neutrophil-targeting effects are studied. This will result in more accurate insights on not only how to tamper pro-inflammatory profile neutrophils, but also promote the pro-resolutive neutrophil population, creating potential novel approaches for NASH treatment.

## Supplementary Information

Supplementary Figures.

## Data Availability

All data generated or analyzed during this study are included in this published article (and its Supplementary Information files).
