# Supplementary material for: Dual role of neutrophils in modulating liver injury and fibrosis during development and resolution of diet-induced murine steatohepatitis
Source: Sci Rep. 2021 Dec 17;11:24194. doi: 10.1038/s41598-021-03679-w (PMC8683497; doi:10.1038/s41598-021-03679-w)
Supplement: Supplementary file 1 — Supplementary Figures. [file 41598_2021_3679_MOESM1_ESM.pdf]

SUPPLEMENTARY INFORMATION

Dual role of neutrophils in modulating liver injury and fibrosis during development and resolution of diet-induced murine steatohepatitis

Andrea D. Kim (adk002@health.ucsd.edu) [1+], Sung Eun Kim (sekim@hallym.or.kr)[2, 3+], Aleksandra Leszczynska (aleszczynska@health.ucsd.edu)[1], Benedikt Kaufmann (bkaufmann@health.ucsd.edu)[1,4], Agustina Reca (areca@health.ucsd.edu)[1], Dong Joon Kim (djkim@hallym.ac.kr)[2, 3], Ariel E. Feldstein (afeldstein@ucsd.edu) [1*].

[1]Department of Pediatrics, University of California San Diego, La Jolla, USA

[2]Department of Internal Medicine, Hallym University College of Medicine, Chuncheon, Republic of Korea

[3]Institute for Liver and Digestive Diseases, Hallym University, Chuncheon, Republic of Korea

[4]Department of Surgery, TUM School of Medicine, Klinikum rechts der Isar, Technical, University of Munich, Munich, Germany

[+]Contributed equally.

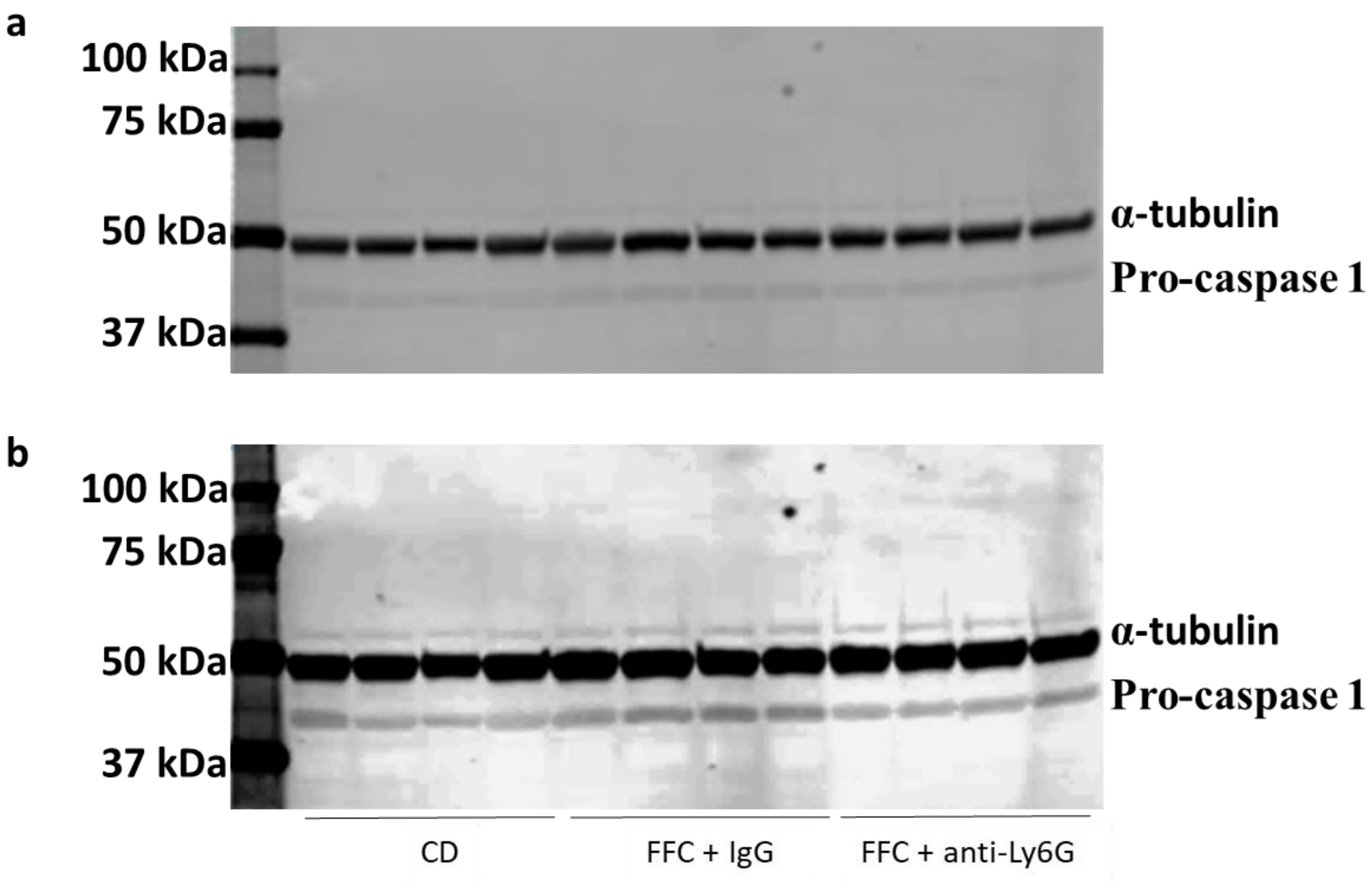

SUPPLEMENTARY FIG. 1: Full length Western Blot for detection of pro-caspase-1 in whole liver tissue of mice fed with Control Diet (CD) or high fat-fructose-cholesterol (FFC) diet in association with either IgG (FFC + IgG) or anti-Ly6G (FFC + anti-Ly6G) antibodies administration. (a) Western blotting for detection of pro-caspase-1 was performed. Active caspase-1 was not visualized, unlike precursor procaspase 1 (MW: 46 kDa). α-tubulin (MW: 53 kDa) was used as housekeeping control. (b) Higher resolution and exposure levels were adjusted for a better visualization of the pro-caspase 1 band.

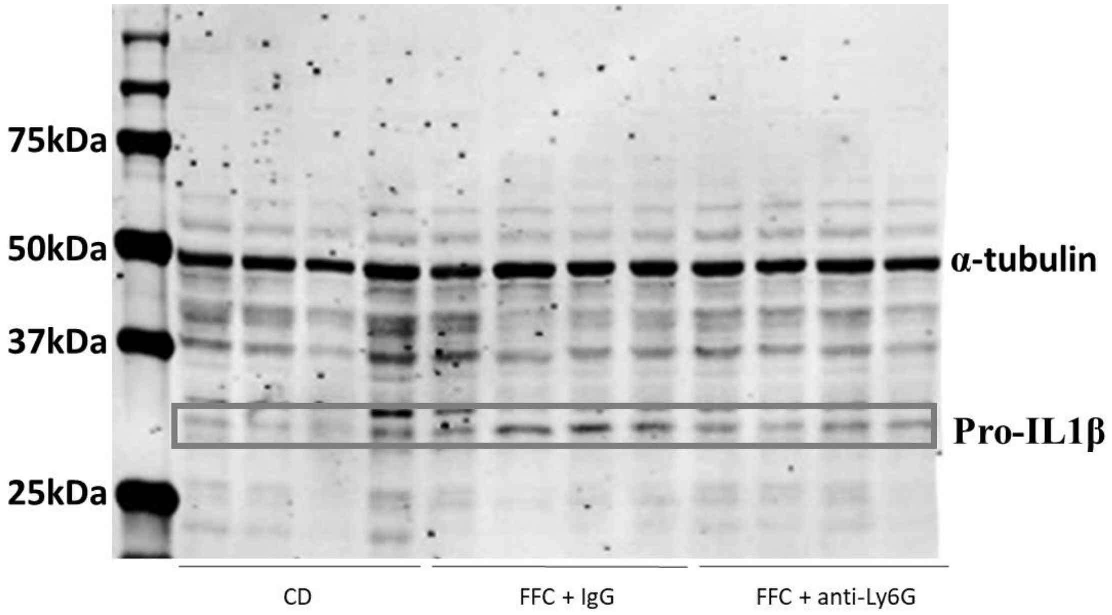

SUPPLEMENTARY FIG. 2: Full length Western Blot for detection of pro-IL-1β in whole liver tissue of mice fed with CD or FFC diet in association with either IgG or anti-Ly6G antibodies administration. (a) Western blotting for detection of pro-IL-1β was performed. Active IL-1β was not visualized, unlike precursor pro- IL-1β (MW: 31 kDa). Detection of α-tubulin (MW: 53 kDa) was used as housekeeping control.

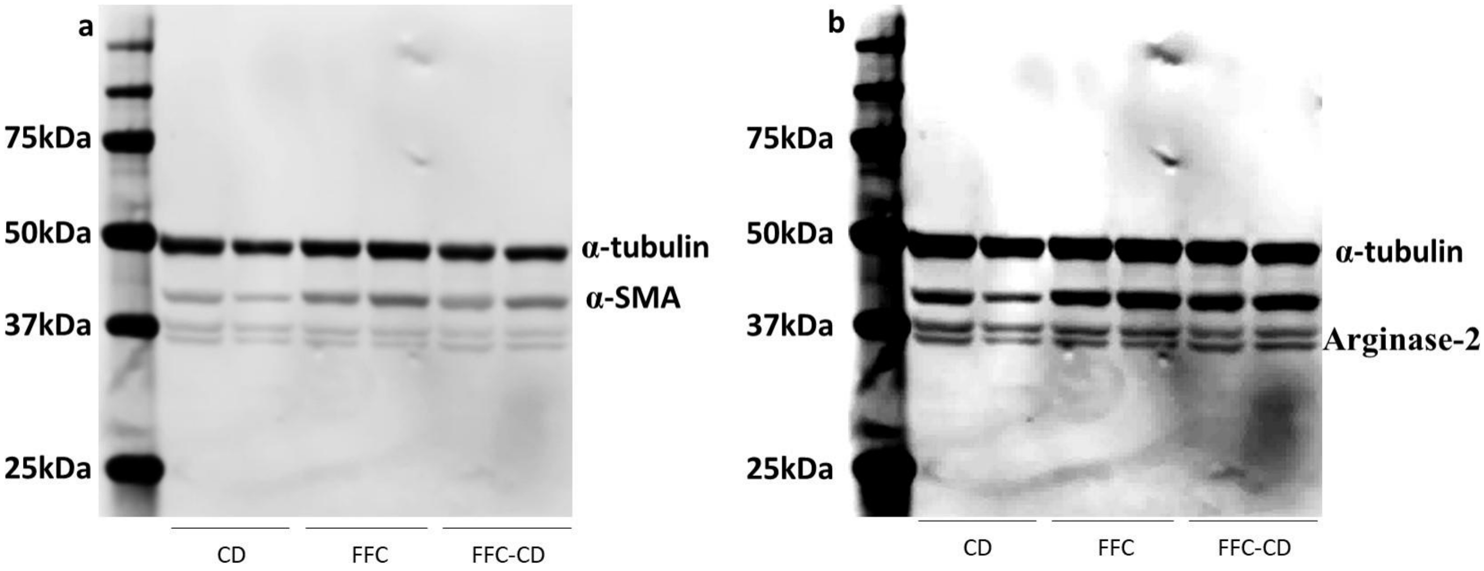

SUPPLEMENTARY FIG. 3: Full-length Western Blot for detection of alpha smooth muscle actin (α-SMA) and arginase-2 in whole liver tissue of mice fed with CD, FFC diet or FFC diet with a reversal into control diet (FFC-CD, allowing spontaneous inflammation resolution). (a) Western blotting for detection of α-SMA (MW: 42 kDa) and arginase-2 (MW: 35 kDa) was performed. Detection of α-tubulin (MW: 53 kDa) was used as housekeeping control. (b) Higher exposure was adjusted for a better visualization of arginase-2 band.

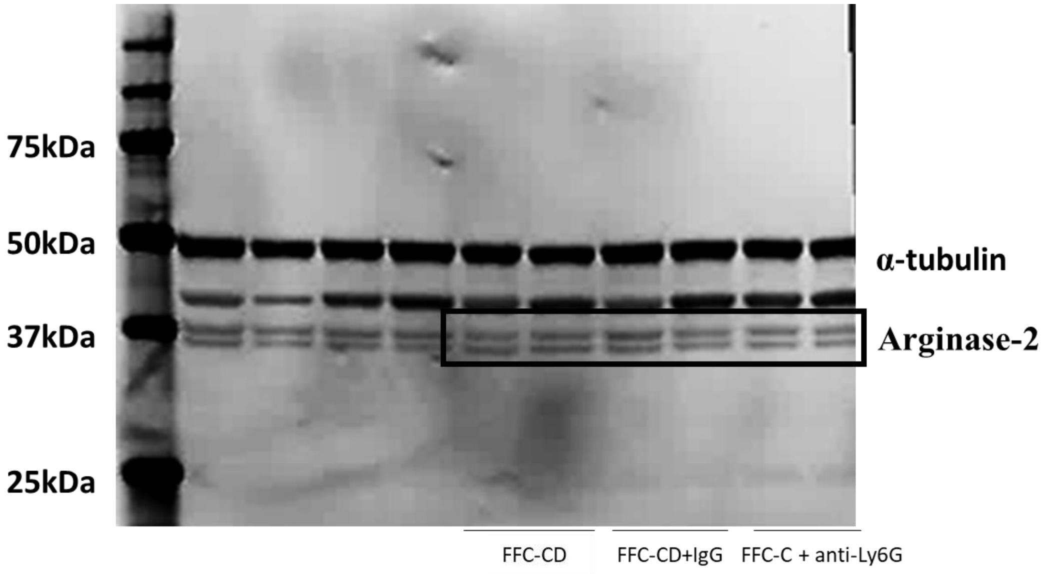

SUPPLEMENTARY FIG. 4: Full-length Western Blot for detection of arginase-2 was performed in whole liver tissue of mice treated with a diet reversal into control diet (FFC-CD, allowing spontaneous inflammation resolution) alone or in association of either IgG (FFC-CD + IgG) or anti-Ly6G antibodies (FFC-CD + anti-Ly6G) administration. (a) Western blotting for detection of arginase-2 (MW: 35 kDa) was performed. Detection of α-tubulin (MW: 53 kDa) was used as housekeeping control.

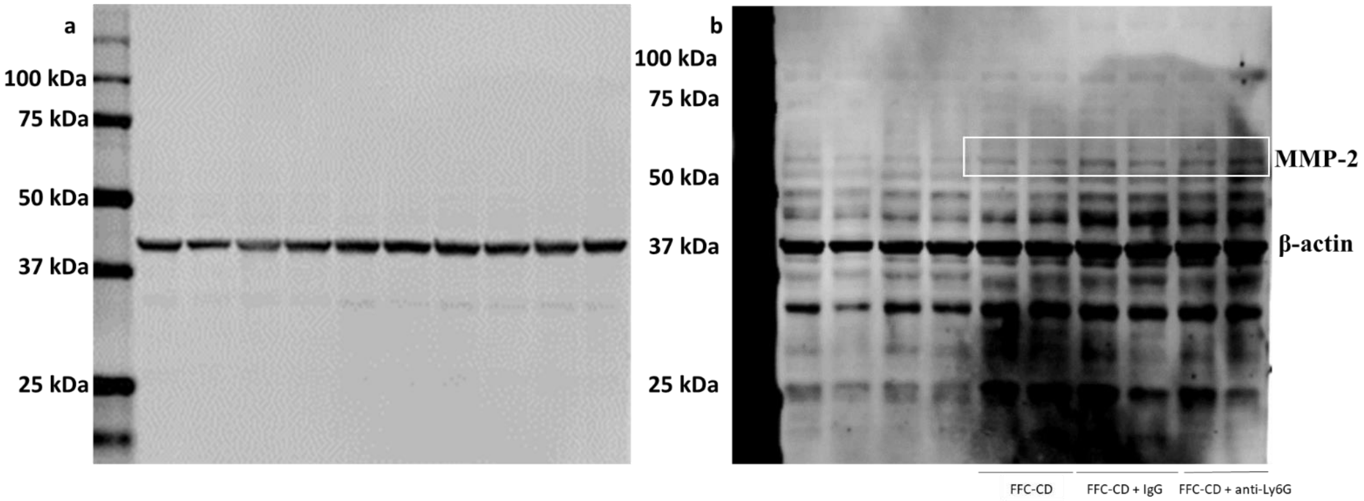

SUPPLEMENTARY FIG. 5: Full-length Western Blot for detection of matrix metalloproteinase-2 (MMP2) in whole liver tissue of mice that underwent diet reversal into control diet alone (FFC-CD, allowing spontaneous inflammation resolution) or in association of either IgG or anti-Ly6G antibodies administration. (a) Western blotting for detection of MMP-2 (predicted MW: 74 kDa) was performed. Detection of β-actin (MW: 43 kDa) was used as housekeeping control. (b) Higher resolution scanning and increased exposure was adjusted for a better visualization of the MMP-2 band.
